# Modeling Mutual Exclusivity of Cancer Mutations

**DOI:** 10.1371/journal.pcbi.1003503

**Published:** 2014-03-27

**Authors:** Ewa Szczurek, Niko Beerenwinkel

**Affiliations:** 1 Department of Biosystems Science and Engineering, ETH Zurich, Basel, Switzerland; 2 SIB Swiss Institute of Bioinformatics, Basel, Switzerland; Weizmann Institute of Science, Israel

## Abstract

In large collections of tumor samples, it has been observed that sets of genes that are commonly involved in the same cancer pathways tend not to occur mutated together in the same patient. Such gene sets form mutually exclusive patterns of gene alterations in cancer genomic data. Computational approaches that detect mutually exclusive gene sets, rank and test candidate alteration patterns by rewarding the number of samples the pattern covers and by punishing its impurity, i.e., additional alterations that violate strict mutual exclusivity. However, the extant approaches do not account for possible observation errors. In practice, false negatives and especially false positives can severely bias evaluation and ranking of alteration patterns. To address these limitations, we develop a fully probabilistic, generative model of mutual exclusivity, explicitly taking coverage, impurity, as well as error rates into account, and devise efficient algorithms for parameter estimation and pattern ranking. Based on this model, we derive a statistical test of mutual exclusivity by comparing its likelihood to the null model that assumes independent gene alterations. Using extensive simulations, the new test is shown to be more powerful than a permutation test applied previously. When applied to detect mutual exclusivity patterns in glioblastoma and in pan-cancer data from twelve tumor types, we identify several significant patterns that are biologically relevant, most of which would not be detected by previous approaches. Our statistical modeling framework of mutual exclusivity provides increased flexibility and power to detect cancer pathways from genomic alteration data in the presence of noise. A summary of this paper appears in the proceedings of the RECOMB 2014 conference, April 2–5.

## Author Summary

Tumor DNA carries multiple alterations, including somatic point mutations, amplifications, and deletions. It is challenging to identify the disease-causing alterations from the plethora of random ones, and to delineate their functional relations and involvement in common pathways. One solution for this task is inspired by the observation that genes from the same cancer pathway tend not to be altered together in each patient, and thus form patterns of mutually exclusive alterations across patients. Mutual exclusivity may arise, because alteration of only one pathway component is sufficient to deregulate the entire process. Detecting such patterns is an important step in *de novo* identification of cancerous pathways and potential treatment targets. However, the task is complicated by errors in the data, due to measurement noise, false mutation calls and their misinterpretation. Here, we propose a fully probabilistic, generative model of mutually exclusive patterns accounting for observation errors, with interpretable parameters that allow proper evaluation of patterns, free of error bias. Within our statistical framework, we develop efficient algorithms for parameter estimation and pattern ranking, together with a statistical test for mutual exclusivity, providing more flexibility and power than procedures applied previously.

This article is associated with RECOMB 2014.

## Introduction

Recent years in cancer research are characterized by both accumulation of data and growing awareness of its overwhelming complexity. While consortia like The Cancer Genome Atlas (TCGA) [1] and the International Cancer Genome Consortium (ICGC) generate the multidimensional profiles of genomic changes in various cancer types, computational approaches struggle to pinpoint its underlying mechanisms [2]. The most basic yet already challenging task is to identify cancer drivers, genomic events that are causal for disease progression. A second, more general task is to elucidate sets of functionally related drivers, such as mutations of genes involved in a common oncogenic pathway.

One systematic approach to address the latter task is to search for mutually exclusive patterns in cancer genomic data [3]–[7]. Typically, the data is collected for a large number of tumor samples, and records presence or absence of genomic alterations, such as somatic point mutations, amplifications, or deletions of genes. In mutually exclusive patterns, the alterations tend not to occur together in the same patient. These patterns are commonly characterized by their coverage and impurity. Coverage is defined as the number of patient samples in which at least one alteration occurred, while impurity refers to non-exclusive, additional alterations (referred to as non-exclusivity or coverage overlap in previous studies). Such mutually exclusive alterations have frequently been observed in cancer data [8]–[10] and were associated with functional pathways or synthetic lethality [3]–[8], [11], [12]. Therefore, mutually exclusive patterns are important for a basic understanding of cancer progression and may suggest genes for targeted treatment.

Previous studies identified mutually exclusive patterns either via integrated analysis of known cellular interactions and genomic alteration data [6], or *de novo*, by an online learning approach [3], or by maximizing the mutual exclusivity weight introduced by Vandin and colleagues [4], [5], [7]. The weight increases with coverage and decreases with coverage overlap [4] and proved successful for pattern ranking and cancer pathway identification.

To our knowledge, there exists no approach that explicitly models the generative process of mutual exclusivity patterns. In the absence of a statistical model of the data, the definition of the weight, although intuitively reasonable, remains arbitrary. In the previous studies, the weight served also as statistic for a column-wise permutation test that assesses the significance of patterns. We show that the power of this test decreases with the number of genes, likely because the weight does not scale with gene number, and the same impurity level affects it more with more genes in the pattern. Most importantly, none of the existing approaches deal with the problem of errors in the data. Despite advanced methodologies on both experimental and computational side [13], records of genomic alterations may contain false positives and false negatives, due to measurement noise, as well as uncertainty in mutation calling and interpretation. As illustrated in Figure S1, ignoring errors in the data, particularly false positives, may lead to wrong ranking of patterns.

**Figure 1:**
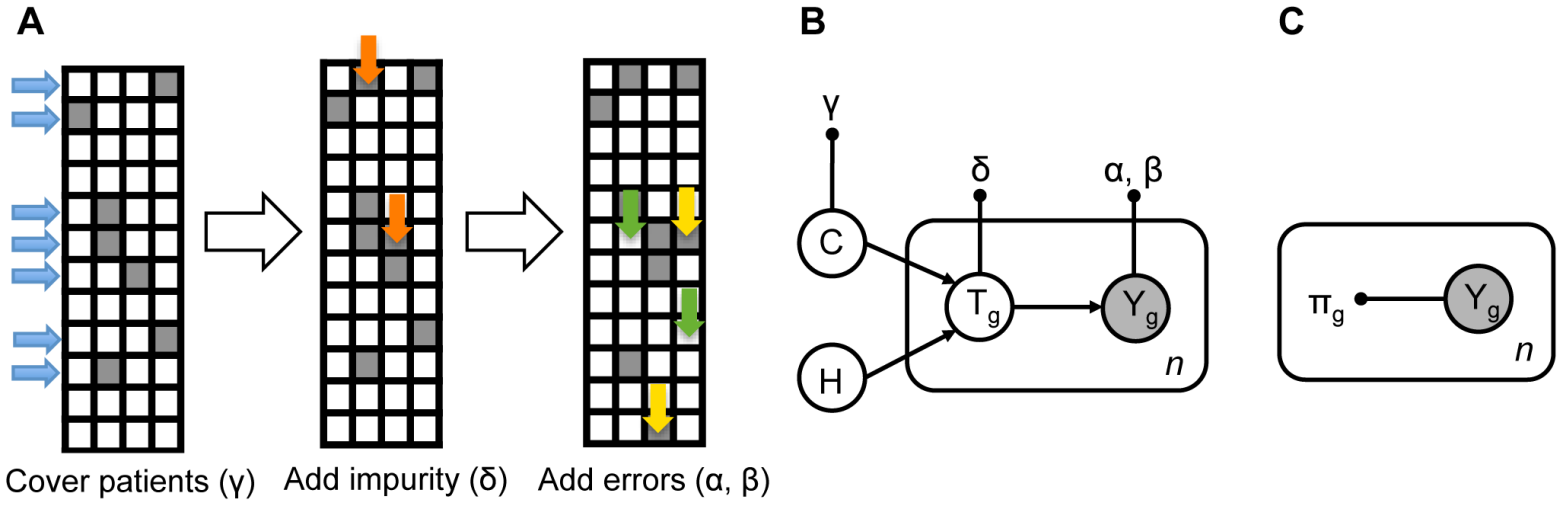
Principles of the mutual exclusivity model and test. **A** The generative process underlying mutual exclusivity patterns. The matrices show alteration status (shaded for presence and white for absence of alteration) for genes (columns) in patients (rows) in consecutive steps of the process, each dependent on parameters indicated in brackets. Blue arrows point at patients that are covered by the pattern with probability 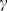. Orange arrows point at impure alterations, added with probability 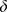. Yellow and green arrows show false positives (added with rate 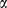) and false negatives (rate 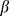), respectively. **B** Graphical representation of the mutual exclusivity model. Large circles: random variables, with observed variables shaded. Small black circles indicate model parameters, and are connected to their corresponding variables with edges. Arrowed edges show dependencies between variables. The rectangle plate indicates a set of identically distributed variables or a set of their parameters (with indices 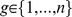). **C** The independence model.

Here, we develop two alternative models for cancer alteration data (Figure 1). One is a probabilistic, generative model of mutually exclusive patterns in the data. The model contains coverage as well as impurity as parameters, together with false positive and false negative rates. We show analytically that the model parameters are identifiable, and propose how they can be estimated and used for pattern evaluation. The second is a null model assuming independent alterations of genes. Via comparison of the mutual exclusivity model to the null model, our approach allows statistical testing for mutual exclusivity, both in the presence and absence of errors.

First, we evaluate performance of our approach in the case when, as it is done in the literature, the data is assumed to record no false positive or negative alterations. On simulated patterns our mutual exclusivity test proves more powerful than the weight-based permutation test. In glioblastoma multiforme data [14], analyzed by the previous approaches, we find novel, biologically relevant patterns, which are not detected by the permutation test. Next, we examine the bias introduced in pattern ranking by ignorance of errors, especially false positives, and show that when the error rates are known, our approach is able to accurately estimate the true coverage and impurity and rank the patterns accordingly. Finally, we analyze the practical limits of accurate parameter estimation in the most difficult, but also most realistic case where the data contains errors occurring at unknown rates. We apply our approach to a large, pan-cancer collection of 3299 tumor samples from twelve tumor types [15], for which the model accounting for the presence of false positives can accurately be estimated. This model is shown to be more flexible than the model assuming no errors in the data, and is applied to identify several universal, significant mutual exclusivity patterns, which would not be found by the previous methods.

## Results

### Modeling and testing for mutual exclusivity

A mutual exclusivity pattern can be detected in a given cancer alteration dataset, with 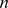 columns that correspond to a subset of measured genes and 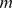 rows (observations) that correspond to patients whose tumor samples were collected (with 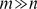). For each patient and gene, the dataset records a binary alteration status of the gene observed in the patient, with 0 standing for absence and 1 for presence of alteration.

We assume that the mutual exclusivity patterns are the result of the following generative process (Figure 1A). First, with a certain probability, denoted 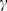 and called coverage, the patients who are covered by the pattern are chosen. Each row corresponding to a covered patient is hit by an exclusive alteration, meaning that exactly one gene is assigned value 1 in this row. Here, we assume that all genes have equal probability to be exclusively mutated. Next, in the same row, with probability 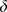, any other gene can be mutated in addition. Those added alterations are interpreted as impurity in the mutual exclusivity pattern, hence 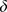 is referred to as the impurity parameter. The generative process described up to this point coincides with the data simulation procedure used in previous studies [4], [5]. However, the corresponding generative model was not used for statistical inference. This prevalent view of the generative process ignores the possible occurrence of errors. Realistically, the observed alteration data result from adding false positives (with rate 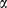) and false negatives (rate 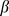) to the true, exclusive, and impure alterations.

We propose a generative model of mutual exclusivity that describes the process illustrated in Figure 1A. For each patient in a given dataset, the proposed model (Figure 1B and Methods) assigns a probability to the corresponding observation. The model is defined by a set of hidden random variables 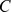, 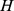, 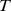, and observed variables 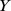. The binary variable 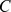 has value 1 with probability 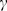, indicating that the patient is covered by the mutual exclusivity pattern. The hidden random variable 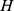 points at the gene that is exclusively altered in that pattern. The set of hidden random binary variables 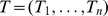 corresponds to the true alteration status of the genes, and the set of observed binary variables 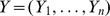 corresponds to the alteration status that is recorded in the data. Each true alteration variable 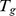 has value 1 either if it was chosen to be exclusively altered, or if it was not chosen but acquired an impure alteration with probability 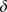. The values of the variables 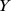 are the same as values of 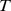, except for cases of false positives (with probability 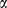) and false negatives (with probability 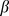). First, we analyzed the identifiability of the model from observed data (Text S1):

**Proposition 1** For 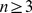, the parameters in the mutual exclusivity model are identifiable.

Encouraged by this result, we propose an expectation maximization algorithm (Methods) to estimate the maximum likelihood parameter values and evaluate its performance in practice (Results).

In the case when the dataset does not carry the mutual exclusivity pattern, we assume that the corresponding genes are mutated independently with their individual alteration frequencies. This is modeled with a set of independent, observed binary random variables 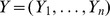, satisfying 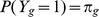 for each 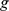 (referred to as the independence model; Text S1). We devise a mutual exclusivity test (shortly, ME test), which compares the likelihood in the mutual exclusivity model to the likelihood in the independence model. Since the models are not nested, we use Vuong's closeness test [16] to compute the p-values (Methods). A small p-value means that the mutual exclusivity model is closer (with respect to Kullback-Leibler divergence) to the true model from which the data was generated than the independence model. The test statistic accounts for the difference in degrees of freedom between the models.

We evaluate our mutual exclusivity model and statistical test in three different scenarios. First, we make an assumption prevalent in the literature, namely that the data is generated without errors. In the second scenario, we assume that the data contains errors, and the error rates are given. Finally, we consider the scenario where the data is generated with errors, and the error rates are unknown.

### Modeling mutual exclusivity patterns without errors

**Figure 2:**
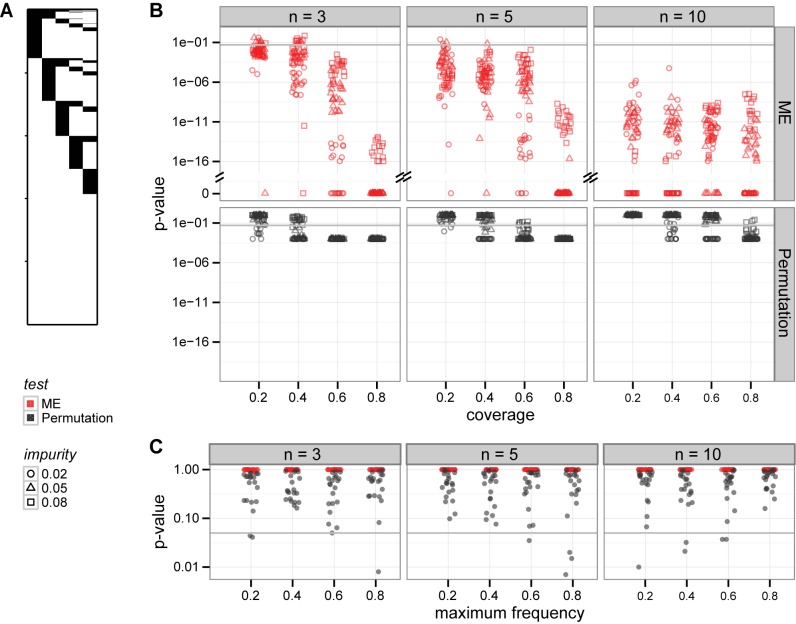
Our mutual exclusivity (ME) test is more powerful than a permutation test, which was applied previously. **A** Example simulated mutual exclusivity pattern. **B** The ME test shows smaller p-values with growing number of genes in the patterns. On the contrary, the permutation test (with 1000 column-wise permutations) is less powerful for larger patterns. **C** Both tests do not support mutual exclusivity in data generated from the independence model.

First, we evaluate the performance of our mutual exclusivity model on simulated data assuming that the data is clean of errors. In this case, the model is reduced, since it is parametrized only by the coverage 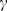 and the impurity 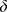, and the observed variables 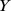 are equated with the true hidden variables 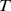. We have derived closed-form expressions for the maximum likelihood parameter values (Methods), providing reliable parameter estimates already for datasets of sample size 200 (Table S1). We simulated datasets from the reduced mutual exclusivity model, for increasing gene set sizes, 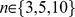, 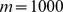 patients, and combinations of parameter values 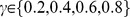 and 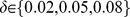, with 20 datasets generated per each parameter setting (example in Figure 2A). For each dataset, we assessed the significance of mutual exclusivity using the proposed ME test (Methods). For comparison, we obtained empirical p-values from the weight-based permutation test, which permutes individual columns in the dataset 1000 times, and reports the number of times a permuted dataset had a higher weight than the original [4], [5].

For datasets with three genes only and low coverages, both our ME and the permutation test not always detect mutual exclusivity (Figure 2B). As the gene set size increases, in contrast to the permutation test, the ME test becomes more powerful. With ten genes, our test supports mutual exclusivity for all datasets, whereas the permutation test does not, even for a large fraction of datasets with high coverage. As an example, for the mutual exclusivity pattern in Figure 2A the ME test p-value is 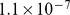, and the permutation test p-value is 0.15. We speculate that the reason for the decreased power of the permutation test is the weight itself. With the same coverage and impurity, large gene sets get less significant weights than small gene sets, since the weight decreases drastically with addition of impure alterations in each row, and this addition is more likely for longer rows. In addition, with increased gene set size the ME test p-values tend to decrease. This suggests that the test will remain powerful also after multiple hypothesis testing correction, which is expected to be more restrictive for larger set sizes.

Both tests correctly do not support mutual exclusivity for datasets generated from the independence model (Figure 2C). 20 datasets were simulated per each maximum individual frequency 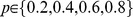 (each frequency 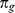 was drawn at random uniformly from interval 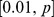). The same, correct behavior was observed when the independent frequencies were drawn from a distribution observed in real cancer data (Figure S2). Figures 2B,C show that the ME test, without computationally expensive permutations, yields ranges of p-values that are amenable to multiple testing corrections. In summary, the ME test is equally powerful for small gene sets as the permutation test, and more powerful for larger ones, and can efficiently be applied in practice.

We further use our model to identify significant mutual exclusivity patterns with high coverage and low impurity in glioblastoma multiforme samples from The Cancer Genome Atlas (TCGA [14]; extended collection; originally published with fewer samples [1]). The data were organized in a binary matrix combining point mutations and copy number variants for 236 patients in 83 genes. The genes and their alterations were selected to represent significant players and events in disease progression (Methods).

**Figure 3:**
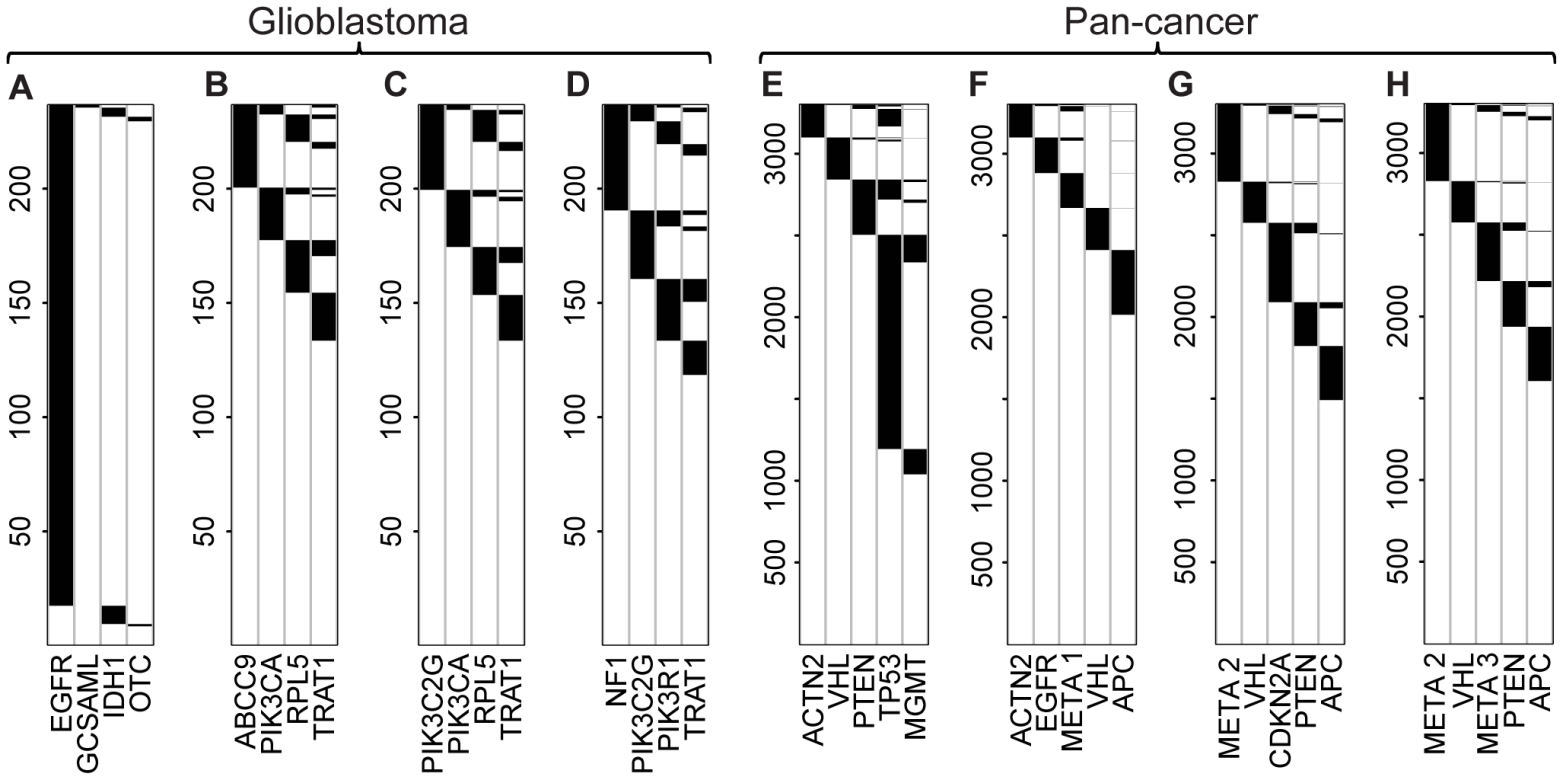
Top mutual exclusivity patterns identified in cancer data. **A–D** Patterns in glioblastoma. **A** Pattern for the gene set with the highest weight (scoring high coverage and low impurity, applied in previous studies), with adjusted permutation test p-value 0. **B–D** Examples of significant, high quality patterns identified using the reduced mutual exclusivity model (assuming no errors), with estimated coverage larger by 0.3 and impurity lower than 0.2. **E–H** Patterns in pan-cancer data. **E** Pattern the for gene set with the highest weight. **F–H** Examples of significant, high quality patterns identified using the mutual exclusivity model that accounts for false positives, with estimated coverage larger by 0.3 and impurity lower than 0.2.

To obtain a comprehensive picture of the types of patterns that can be found in this dataset, we restricted the gene set size to four, and evaluated all 1,837,620 possible gene subsets of this size. Figure 3A presents the pattern with the largest weight, but also large imbalance: in that pattern, almost the entire coverage comes from alterations of a single gene, *EGFR*. With our approach the quality of each pattern can be assessed with the estimated coverage and impurity parameters, while its significance is given by the p-value from the ME test. In the standard understanding, a high quality pattern has high coverage and low impurity. For the GBM dataset we obtained 11 significant (Benjamini-Hochberg adjusted ME p-value 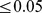) patterns with estimated coverage larger than 0.3 and impurity lower than 0.2 (Table S2). Figure 3B–D presents top three of those patterns with the lowest impurity. Out of the genes included in those top sets, *NF1*, *PIK3C2G*, *PIK3R1* and *PIK3CA* play roles in the interconnected canonical glioblastoma signaling [1], although are not found directly grouped into individual pathways as identified by the original publication. Notably, the TRAT1 protein is a known interaction partner of PIK3R [17], [18].

Table 1 summarizes the statistics for all presented patterns, underlining the differences between the ME and permutation tests. With the explicit account for coverage and impurity as parameters in the model, our approach gives control over which important features of the patterns should be used to prioritize the significant patterns of interest. In contrast to the permutation test, the ME test is specifically designed to prefer balanced patterns. Consequently, patterns identified using our ME approach have over three times lower median imbalance than the median imbalance of top weight patterns with adjusted permutation test p-values 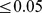 (Figure S3). To assess the imbalance of a given gene set, we calculated the ratio between the number of alterations of the gene with the largest individual frequency in the set to the total number of patients covered with the pattern.

**Table 1:** Summary of top patterns identified for the glioblastoma dataset assuming no errors.

Gene set	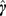	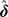	ME statistic	ME p-value	Weight	Perm. p-value	Imbalance
*EGFR*, *GCSAML*, *IDH1 OTC*	0.97	0.01	−14.72	1	221	0	0.93
*ABCC9*, *PIK3CA*, *RPL5 TRAT1*	0.44	0.11	2.63	0.04	69	1	0.28
*PIK3C2G*, *PIK3CA*, *RPL5 TRAT1*	0.44	0.11	2.84	0.02	68	1	0.27
*NF1*, *PIK3C2G*, *PIK3R1*, *TRAT1*	0.5	0.13	2.63	0.04	72	1	0.28

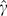
: estimated coverage; 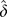: estimated impurity; ME statistic: Vuong statistic comparing the likelihood under the mutual exclusivity model to the likelihood under the independence model; ME p-value: p-value in the ME test (Vuong's test with ME statistic; adjusted using the Benjamini and Hochberg method). Weight: a mutual exclusivity score, applied in previous studies, which increases with coverage and decreases with impurity; Perm. p-value: adjusted p-value in a weight-based column permutation test. Imbalance: contribution of the most frequently mutated gene in the pattern to the coverage.

Our analysis did not rediscover four mutually exclusive gene sets (Table S3) identified previously based on optimizing the weight [4], [7] for the first, original GBM dataset version. Several genes in those sets did not pass our filtering criteria in the pre-processing step (Methods), and one gene set could not be analyzed for this reason. Two sets had large estimated impurity(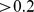), which does not satisfy our threshold. All three analyzed gene sets were insignificant according to the ME test, most likely due to relatively high imbalance (two to three times larger than median imbalance of gene sets we identified, compare Figure S3). Interestingly, one of those gene sets does not have a significant permutation p-value, which may be due to the fact that the processing of the data was different and the original dataset contained fewer samples.

### Modeling mutual exclusivity with known error rates

In this section, we consider the scenario where the data are erroneous, and the error rates are known and can be used for pattern evaluation. Figure S1 visualizes the severe effects of error ignorance. The observed weight, computed on datasets with false negatives, is consistently reduced as compared to the true weight of patterns generated without errors. Addition of false positives introduces most bias in the observed weight, and results in false ranking. Similarly, for the reduced mutual exclusivity model assuming no errors, parameter estimation fails in the case when they do occur in the data (Figure S4). Thus there is a well motivated need for the model to account for errors.

**Figure 4:**
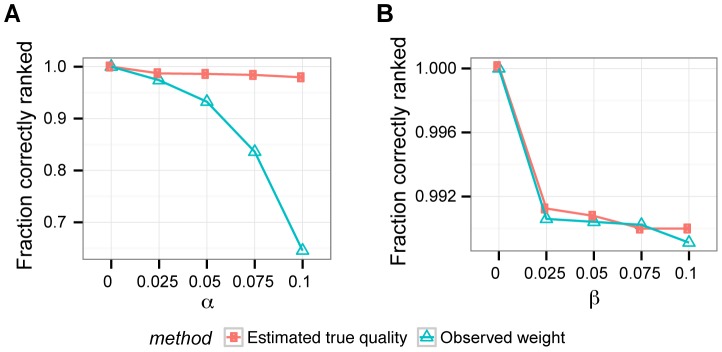
Improved ranking of erroneous patterns. In contrast to the observed weight, which was applied in previous studies, and ignores errors and scores observed coverage and impurity, our approach to estimate true quality, using known error rates, estimates the true parameters and ranks the patterns correctly. The data was simulated from the mutual exclusivity model with parameter values fixed to 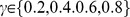, 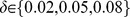, with error rates **A** 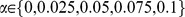 (x-axis), 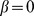, as well as **B** 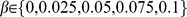 (x-axis), 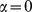. 20 datasets with 5 genes and 1000 patients were simulated per each parameter setting.

Fixing the parameters 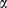 and 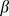 in our model to the true false positive and negative rates, respectively, we can estimate the remaining coverage 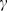 and impurity 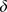 parameters using the EM algorithm (Methods). This estimation is very precise for simulated datasets with five genes, and sample sizes 200 or 1000 (Table S1, Figure S5). Figure 4 shows that such precise estimates can be used to rank the patterns by their estimated true quality, first sorting by the estimated impurity and second by their estimated coverage. We ranked the erroneous datasets simulated in Figure S1 by their estimated true quality. Next, we evaluated the fraction of dataset pairs which were ordered the same way as when their true impurity and coverage were used for sorting. This fraction of correctly ranked pairs was compared to the fraction that is ranked the same way by the observed weight as compared to the true weight. For data containing false negatives both the quality ranking and the observed weight perform very well in correct ranking. The estimated true quality significantly outperforms the observed weight in the presence of false positives.

### Modeling mutual exclusivity with unknown error rates

Finally, we consider the scenario, where the observed data contains errors that occur at unknown rates. In this case we need to estimate all four model parameters, and we proved the model to be identifiable from the data (Text S1). As expected, Table S1 shows that for realistic sample and gene set size (200 or 1000 patients and five genes), and for typical parameter settings (with small impurity 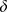 and error rates 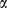 and 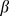), parameter estimation is more difficult than in the case where 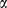 and 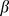 are given (compare Figure S5). The estimated parameter values start approaching the true ones only for prohibitively large sample sizes (Figure S6). In particular, for realistic sample numbers, the parameter 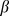 is largely underestimated. Since in case of mutual exclusivity and small 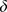 values, there are in total not many true positive cases, the actual false negatives should be very rare. Thus, without much loss of generality of our approach for realistic datasets, we further assume that the false negative rate 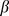 is zero, and account only for the false positives. With this assumption, our approach is still very useful in mutual exclusivity analysis: Figure S1 and Figure 4 show that in terms of ranking there is a pressing need to account for the false positives rather than for false negatives.

**Figure 5:**
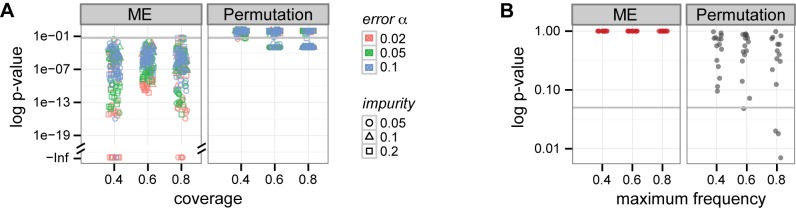
Power of the mutual exclusivity model accounting for false positives. The ME test p-values for **A** data generated from the full mutual exclusivity model with 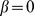 given, and **B** generated from the independence model, in comparison to a permutation test applied in previous studies. Again, the ME test is more powerful (compare Figure 2).

Table S1 and Figure S7 illustrate that with this assumption, already for 1000 samples (but not 200) a much more accurate estimation of the remaining parameters 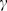, 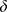, and 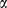 is possible. Still, for impurity 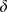 too similar to false positive 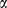, the 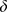 parameter is overestimated, and 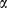 underestimated. Thus, in some cases, the true impurity 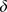 may be smaller than its estimated value, making our evaluation of patterns over-conservative. Again, this problem diminishes for larger datasets. Figure 5 shows, that for realistic dataset sizes and parameter sizes, the ME test is able to detect mutual exclusivity in data with false positives, and is more powerful than the permutation test.

We applied our approach accounting for false positives to pan-cancer genomic alteration data [15], a data collection from twelve distinct cancer types. Combining cancer datasets enables to mine for mutually exclusive patterns that are universal for the disease, but can be a problem for the search of patterns that are specific for one of the combined types. A gene set which has mutually exclusive alterations in only one cancer type and not others will most likely not be detectable in the combined dataset. The pan-cancer dataset is much larger than the glioblastoma data, thus allowing more accurate parameter estimation. Somatic point mutations, copy number variants, and methylations were compiled into a single binary data matrix. Duplicated columns from the compiled matrix were removed, yielding a matrix with 428 columns, some of which represent not one, but several genes (Methods).

We aimed to collect universal, low-impurity mutual exclusivity patterns for gene sets of size five that cover multiple cancer samples, accounting for possible false positives. We first pre-filtered the immense set of all possible subsets, starting with fitting the reduced model (assuming no errors in the data) for all 15,504 subsets of 20 measured genes that were selected by their large individual alteration frequency (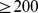; c.a. 0.6%). Next, we chose the 2039 subsets that had estimated coverage larger than 0.3, impurity lower than 0.2, and ME statistic larger than 0, indicating the reduced mutual exclusivity model fits the data better than the independence model (not necessarily significantly). Figure 3E shows the pattern that in the pre-filtered dataset has the largest weight, which is largely dominated by alterations of TP53. Finally, we applied the model accounting for false positives to the pre-filtered subsets, and identified 476 high quality patterns (Table S5) with estimated coverage larger than 0.3, impurity lower than 0.2, selecting by significance (Benjamini-Hochberg adjusted ME p-value 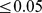), and sorting by impurity (lowest on top; examples in Figures 3F–H). Three out of all columns in the visualized patterns correspond not to one, but a set of genes, and are denoted *META 1-3* (see Table S4 for individual genes). A possible reason for a large number of significant and high quality gene sets (Table S5) is the fact that the identified gene sets overlap. Such overlapping gene sets may either share strongly mutually exclusive subsets of smaller size, or may all be subsets of a single, larger mutually exclusive gene set.

Findings for various cancers for pairs of genes support that the top patterns are indicative of coexistence in a common cancer pathway. For instance, for the pattern in Figure 3G, the protein products of the genes *PTEN* and *MYC* (element of *META 2*) are co-regulators of p53 in control of differentiation, self-renewal, and transformation in glioblastoma [19]. The gene copy ratio of *MYC* and *CDKN2A* in the same pattern has a prognostic value in squamous cell carcinoma of the head and neck [20]. Finally, *PTEN* and *VHL* are both known regulators of the HIF-1 pathway [21]. *PTEN* and *APC*, common to two identified gene sets, are tumor suppressors that are known to interact in cancer [22].

Table S6 compares the p-values and estimated parameters, obtained for the top identified patterns, using the model accounting for false positives to the reduced model. As a rule, the former p-values are smaller, while the values of the coverage and impurity parameters estimated by the two models are similar. In one case however (Figure 3G), the estimated false positive rate is 0.037, yielding the estimated coverage accordingly smaller (0.45) than the estimate from the reduced model (0.55). This is why this pattern, although with larger observed coverage, in our true quality ranking would score lower than the pattern in Figure 3H. In general, for all pre-filtered subsets the ME test based on the model that accounts for false positives was more flexible, and returned a larger number of significant p-values (1397; adjusted ME p-value 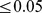), than the test based on the reduced model (1171).

## Discussion

This work brings two main contributions. First, a probabilistic, generative model of mutual exclusivity, with readily interpretable parameters that represent pattern coverage and impurity, as well as parameters that account for false positive and false negative rates. In the case when the data is clear of errors, we give closed-form expressions for maximum likelihood coverage and impurity estimates. For erroneous data, we propose an EM algorithm for parameter estimation. We prove analytically that the model parameters are identifiable, and show the limits of parameter estimation in practice, where the sample sizes are small. These limits allow accurate estimation of the most troublesome false positive rate, as well as the coverage and impurity parameters, which are most useful for pattern ranking. Second, we develop the ME test, which assesses the significance of mutual exclusivity patterns by comparing the likelihood of the dataset under the mutual exclusivity model to the null model assuming independent alterations of genes. The proposed test proves to be more powerful than a permutation test applied previously.

Our approach was first applied to identify mutually exclusive patterns that are specific for glioblastoma, with the assumption prevalent in the literature that the data does not contain errors. The genes that show the top identified patterns are involved in canonical glioblastoma signaling pathways, with addition of two novel genes, *RPL5* and *TRAT1*. Next, we applied the model that accounts for false positives, and detected universal patterns with high coverage and low impurity, found significant by the ME test across a collection of samples from twelve different cancers. Although both these cancer cohorts were already analyzed in detail with cutting-edge tools [1], [3]–[7], [15], our new testing procedure provides new, significant, and biologically relevant patterns that were not identified previously.

The proposed mutual exclusivity model could be extended in several ways. For instance, the current model explicitly assumes that the mutually exclusive mutations occur equally likely in all genes in the dataset. This assumption has two important advantages. First, the ME test finds most evidence for mutual exclusivity for balanced patterns, where the genes contribute similarly to the coverage. Second, with this assumption our EM algorithm is very efficient (Methods) and dropping it would increase its time complexity. The model may be extended to allow different mutually exclusive mutation rates of genes as parameters, which would be estimated from the data. Another possible extension of the model would allow for multiple gene sets, each with own coverage and impurity parameters, and the same error rates. Such a model, in contrast to previous work in this direction [7], would correct for errors and prioritize patterns with balanced mutually exclusive mutations. Finally, this work, focusing on modeling, evaluation, and testing for mutual exclusivity, does not deal with efficient search for mutual exclusivity patterns. Instead, we browse all possible, small gene subsets measured in glioblastoma, or all gene sets with high coverage in the pan-cancer data. Integration of the model into existing [4], [5] or a new search procedure is one direction of our future research. Ideally, the objective optimized in the search would be a single measure that reflects preferred impurity, coverage, and significance in the ME test. These three evaluation criteria could be combined using appropriate priors in the ME model. The results presented here indicate that already now, the proposed approach is a step forward in the demanding task of mining cancer genomic data for the mechanistic principles of this disease.

## Materials and Methods

### Preprocessing of genomic cancer data

The TCGA provisional glioblastoma data for 236 patients in 83 genes includes somatic point mutations (identified as significant by MutSig [23]), amplifications and deletions (called by GISTIC [24]). The combined analyzed dataset is filled with zeros, and has entry 1 whenever there was a significant point mutation, or a copy number variant that is concordant with expression in the data. For each gene, concordance of its copy number variants (amplifications and deletions) with expression data was assessed using the Wilcoxon test, comparing medians of the gene expression in the samples with the variant to expression in diploid samples. Specifically, amplifications were tested to have expression median higher, and deletions to have the median lower than the diploid cases. Only significantly concordant (p-value 0.05) variants were recorded in the analyzed dataset. The pan-cancer TCGA data has 3299 samples and records somatic point mutations, amplifications, deletions and methylations. Pre-processed data was downloaded from the cBioPortal [25] and combined into a single binary matrix with altered genes as columns, separately for the GBM and for the pan-cancer data collection. In the combined pan-cancer matrix some columns were identical, with different genes having alterations in exactly the same patients. Since such genes are undistinguishable with respect to mutual exclusivity patterns, they were combined into “meta” sets of genes, and represented with a single column in the matrix.

### Generative mutual exclusivity model

Let 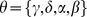 be the set of model parameters, with coverage 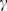, impurity 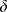, false positive rate 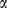 and false negative rate 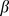. We define the mutual exclusivity model on a set of random variables: hidden binary random variable 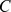 that indicates patient coverage, hidden binary vector variable 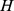 that specifies the single exclusively mutated gene in a covered patient, a set of hidden binary variables 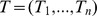 that represent the true alterations of genes, and a set of observed variables 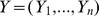 that correspond to the alteration status of genes recorded in the data. The model is defined by:
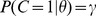


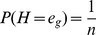


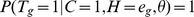


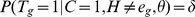


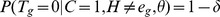


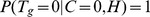


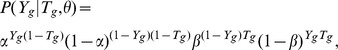
for all 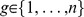, where 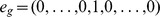 is a unit vector of length 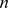 with a single entry 1 at position 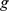. Thus, 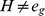 means that some other gene than 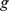 is selected as mutually exclusively mutated. With this distribution of 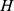, our model is tailored for balanced patterns, where the mutually exclusive alterations occur on average equally frequently for each gene in the pattern. The set of of hidden binary random variables 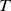 indicates true alterations in the genes. 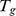 has value 1 either when gene 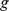 is selected as mutually exclusive (for 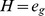), or, otherwise, when the entry for gene 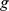 is impure, and it was mutated in addition to another gene (for 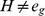). In this model, the observed likelihood 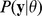 for a given observation 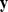 depends only on the number of values 1 in the observation, denoted 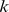, and observation length 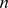, and is thus denoted 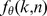 (Text S1). For 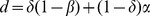 we have:
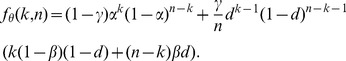
(1)The likelihood of the whole dataset 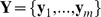 reads:
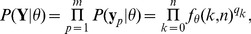
(2)where 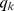 is the number of observations with 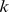 values 1 in 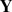. Thus, after pre-computation of 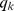 values in 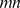 steps, the likelihood can be computed efficiently in only 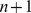 steps of constant time complexity.

#### Parameter estimation in the model without errors

In the reduced model we know 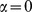 and 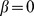 and we are interested only in estimating 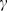 and 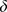. In this case, 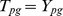 for all 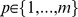, 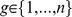, and the log likelihood reads
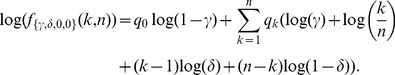
(3)The maximum likelihood parameter estimates are given by 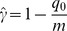 and 
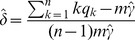
.

#### Parameter estimation in the model with errors

By Proposition (1), we have that for 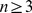 the parameters in the full model are identifiable (Text S1). For maximum likelihood estimation, we propose an EM algorithm (Box 1 and Text S1). In our analysis, we set the input arguments to 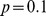, 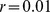, 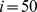, 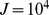 and 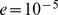. The algorithm utilizes the estimates of the 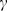 and 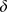 parameters from the reduced mutual exclusivity model (assuming no errors) as educated guesses for initialization. In the E-step, five expected values are computed in constant time for 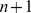 values of 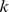. One reason for this computational efficiency is the assumption that 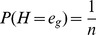 (Text S1). The M-step is performed in constant time. After 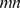 initial pre-computing steps, computation of 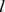 is only 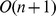, and therefore the complexity of the entire algorithm is 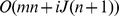. We expect that, as for all mutually exclusive patterns so far observed in the literature, 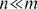 holds. Thus, our algorithm gives a significant reduction in run time of EM in the usual case, where computations need to be performed for all observations, and where 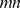 would replace 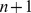 in the complexity. Increasing difficulty of the estimation problem (from both error rates given to unknown, Table S7) for the same 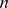 and fixed 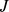, increases the run time, due to larger number of iterations performed (from 21 to 1033 on average). In the case where the data is generated with errors, and the error rates 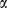 or 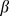 are known, we use the same EM algorithm for estimating the remaining parameters but fix the given values in the M-step.

**Box 1.** EM for Mutual Exclusivity Model.

**Input:** initialization parameters 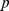, 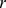, 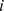,
 iteration parameters 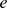, 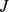;
**Output:** Parameter estimates 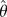

 Estimate 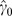 and 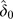 from the reduced mutual exclusivity model
 Draw at random 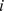 initial parameter settings: 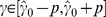,
 

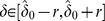
, 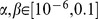

 

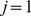
; 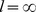;
 
**while** 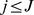 & 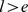
{
    **E-step: for** 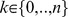

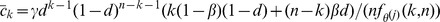

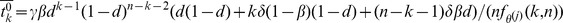

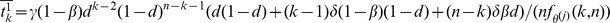

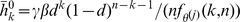

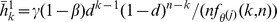

    **M-step:**

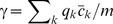

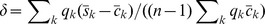

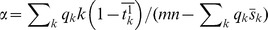

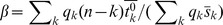

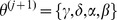

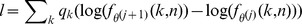

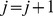

 }
 

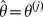

#### Independence model

The independence model assumes all genes are mutated independently. Each gene 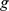 has individual alteration probability 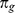, and the vector 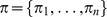 parametrizes the model (Figure 1C). Let 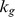 denote the number of patients with alteration in gene 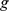. With log likelihood
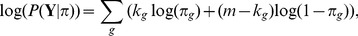
(4)the maximum likelihood parameter values are given by 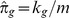.

#### Testing for mutual exclusivity

The mutual exclusivity and independence models are not nested. To compare their likelihoods for a given dataset 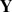, we compute the Vuong's statistic [16] 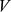, defined by the standardized and corrected log-likelihood ratio:
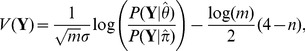
(5)where 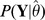 (equation 1) and 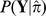 (equation 4) are the observed log likelihoods of the data 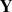 for the maximum likelihood parameter estimates 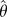 and 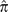 under the mutual exclusivity and independence model, respectively, and 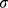 is the standard deviation of the log likelihood ratios across observations. The second term is a correction for the difference in the numbers of free parameters in the models. For non-nested models [16], their 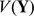 has normal distribution 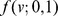 with mean 0 and variance 1, and equals 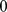 when the models have equal Kullback-Leiber divergence from the true model generating the data 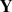. Thus, the ME test p-value is given by 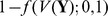.

### Compared methods

For a given set of genes 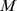, the mutual exclusivity weight [4] is defined as
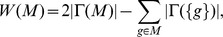
where 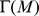 is the number of samples with at least one alteration in 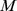. To assess significance of the weight, a permutation test is performed with the weight as test statistic, and the null distribution is obtained by independently permuting alterations 1000 times for each gene (each column in the dataset), preserving its alteration frequency.

A summary of this paper appears in the proceedings of the RECOMB 2014 conference, April 2–5 [26].

## Supporting Information

Figure S1 **Computation of mutual exclusivity weight can be severely biased by errors in the data.** Left plot: mutual exclusivity weight, proposed by Vandin and colleagues [4], for datasets simulated from the mutual exclusivity model without errors. In this case, the observed weight (weight computed on observed data) is the same as the true weight (weight computed on true data, i.e., with true alteration status recorded), and increases with coverage and decreases with impurity. Arrow points at one example pair of datasets, indicating how they are ranked by the true weight. Middle: addition of false negatives decreases the observed weight (here, computed on the observed, erroneous dataset, and not based on the true alteration status), but has a consistent effect and does not disturb the ranking. Right: addition of false positives has most severe effect on ranking using the observed weight. An arrow points at two datasets, which based on the true weight (i.e. computed on data recording true alteration status, as in the left plot) were ordered increasingly, and which are now reverse-ordered by the observed weight.
(PDF)

Figure S2
**Both our mutual exclusivity (ME) test and a permutation test, which was applied previously do not support mutual exclusivity in data generated from the independence model with independent frequencies distributed as in the glioblastoma dataset.** Shown are log p-values for simulated data with 1000 patients, 20 datasets per each gene set size (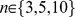).(PDF)

Figure S3
**Imbalance of patterns identified with the ME approach is much lower than of patterns identified using the previously proposed weight.** Box-plots summarize the imbalance distribution for 11 patterns called significant with ME p-value 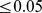, high coverage (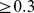) and low impurity (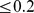; red), as well as the 10, 100, and 1000 top patterns with the largest weight, called significant with permutation test (p-value 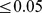). Median imbalance of patterns prioritized using our approach is around three times lower than of patterns with top, significant weights, regardless of how many of the top ones are considered.(PDF)

Figure S4 **Parameter estimation in the reduced mutual exclusivity model can be severely biased by errors in the data.** Left column: the difference between the true and the estimated parameter values for datasets simulated from the mutual exclusivity model without errors. In this case, both impurity (delta; top) and coverage (gamma; bottom) estimation is very accurate, regardless the impurity (marked with colors). The true coverage values are indicated on the x-axis. Middle column: addition of false negatives results in underestimation of the coverage parameter. Right column: addition of false positives results in underestimation of both the impurity and coverage parameters, and most strongly affects estimation of low coverage values.
(PDF)

Figure S5
**Efficient parameter estimation of the coverage parameter** 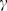 **and the impurity parameter** 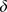 **, using the EM algorithm, from data generated from the mutual exclusivity model with error rates that were given to the model.** The tested true parameter values were fixed to 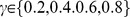, and 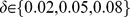 (20 datasets with 5 genes and 1000 patients were simulated per each parameter setting). There are different box plots of estimated parameter values for different true values. The medians of the estimated values are close to the true values, marked with red dashed lines.(PDF)

Figure S6
**Difficulties in estimating the full set of parameters.** We applied our EM algorithm to estimate the coverage parameter 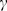, the impurity parameter 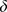, as well as false positive 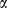 and false negative rate 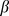, from data generated from the mutual exclusivity model with error rates that were not given to the model, using increasing sample size. The tested parameter values were fixed to realistic values 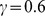, 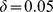, 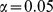 and 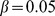. 20 datasets with 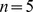 genes and 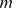 from 1000 (1 K) to 100000 patients (100 K) were simulated. Estimation accuracy increases with sample size.(PDF)

Figure S7
**More accurate parameter estimation assuming false negative rate** 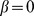 **.** **A** Estimation of parameters 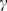, 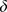, and 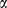 from data generated from the mutual exclusivity model accounting for false positives (false positive rate was not given to the model). The tested parameter values were fixed to 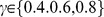, 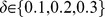, and 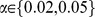. **B** The estimation is more difficult when 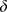 and 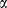 are similar (for 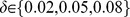). **C** Similarity of 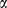 and 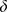 is less of a problem for larger gene sets (here, 10 genes), as well as when more samples are used (not shown). All plots: results on simulations of 20 datasets with 5 genes and 1000 patients per each parameter setting.(PDF)

Table S1
**Root mean squared error (RMSE) of parameter estimation for different model variants and sample sizes.** To determine a reasonable dataset size for the different model variants, we tracked the RMSE of parameter estimates for sample sizes 200 and 1000, with typical parameter settings: 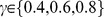, 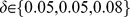 and error rates as indicated in the column “True error rates”. 20 datasets with 5 genes and the number of patients indicated in column “
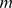
” were simulated from the models per each parameter setting. RMSE was chosen to represent the difficulty of the estimation task as a function of the sample size. For example, for the reduced model that assumes no errors, we have derived closed-form expressions for the maximum likelihood parameter values. Thus, in this case, RMSE of parameter estimates depends only on random variation in the data and defines the best you can get reference for the remaining models, where parameter estimation is more difficult and performed using EM. Since both the ME model likelihood and the test largely depend on how accurately the parameters are estimated, RMSE defines the applicability of the approach.(PDF)

Table S2 **List of high quality, significant gene sets of size four identified in the GBM dataset.**
(TXT)

Table S3 **Results for mutually exclusive patterns identified in the glioblastoma dataset by previous studies.** Analyzed genes are written in bold, to distinguish from genes that were filtered out in preprocessing steps. Publication: the study in which the gene set was identified as mutually exclusive. Other results are given as in Table 1. *from this gene set, only TP53 passed the pre-filtering step, and thus no results are available.
(PDF)

Table S4 **Sets of genes that had identical columns in the combined pan-cancer data matrix and their short names used in the main text.** Genes with identical columns in the combined and binarized pan-cancer data matrix were merged into sets and represented by a single column. The table lists those merged gene sets that are involved in top mutually exclusive patterns identified for the pan-cancer data.
(PDF)

Table S5 **List of high quality, significant gene sets of size five identified in the pan-cancer dataset.**
(PDF)

Table S6
**Summary of top patterns identified for the pan-cancer dataset assuming false positives.** 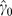, 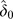, 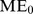 p-value: coverage and impurity estimates, and the p-value from the reduced mutual exclusivity model, assuming no errors in the data. 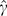, 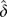, 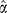, ME p-value: parameter estimates and p-value from the mutual exclusivity model accounting for false positives.(TXT)

Table S7 **Average runtime of the EM algorithm in CPU seconds.** The table presents average runtimes of parameter estimation using the EM algorithm averaged over the datasets simulated and summarized in Table S1. The runtime increases with the difficulty of the parameter estimation problem.
(PDF)

Text S1 **Supplementary Methods.** Likelihood in the mutual exclusivity model, identifiability of the mutual exclusivity model, and derivation of the Expectation Maximization algorithm.
(PDF)
