# Supplementary figures and images for: Modeling Mutual Exclusivity of Cancer Mutations
Source: PLoS Comput Biol. 2014 Mar 27;10(3):e1003503. doi: 10.1371/journal.pcbi.1003503 (PMC3967923; doi:10.1371/journal.pcbi.1003503)

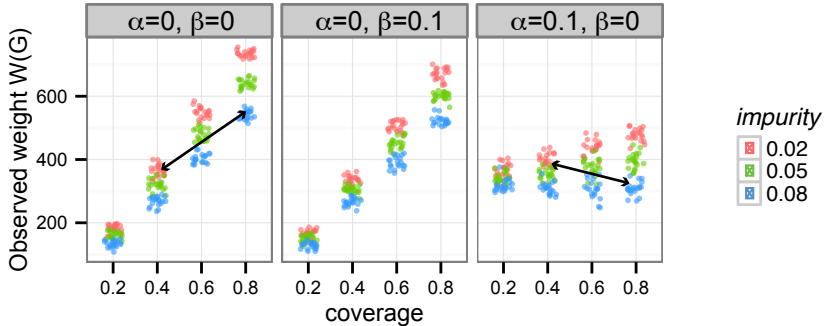

Supplement: Figure S1 — Computation of mutual exclusivity weight can be severely biased by errors in the data. Left plot: mutual exclusivity weight, proposed by Vandin and colleagues [4], for datasets simulated from the mutual exclusivity model without errors. In this case, the observed weight (weight computed on observed data) is the same as the true weight (weight computed on true data, i.e., with true alteration status recorded), and increases with coverage and decreases with impurity. Arrow points at one example pair of datasets, indicating how they are ranked by the true weight. Middle: addition of false negatives decreases the observed weight (here, computed on the observed, erroneous dataset, and not based on the true alteration status), but has a consistent effect and does not disturb the ranking. Right: addition of false positives has most severe effect on ranking using the observed weight. An arrow points at two datasets, which based on the true weight (i.e. computed on data recording true alteration status, as in the left plot) were ordered increasingly, and which are now reverse-ordered by the observed weight. (PDF) [file pcbi.1003503.s001.pdf]

*test*

ME

Permutation

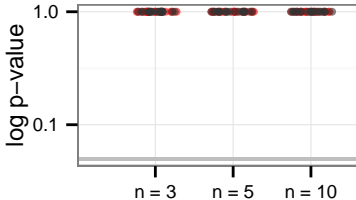

Supplement: Figure S2 — Both our mutual exclusivity (ME) test and a permutation test, which was applied previously do not support mutual exclusivity in data generated from the independence model with independent frequencies distributed as in the glioblastoma dataset. Shown are log p-values for simulated data with 1000 patients, 20 datasets per each gene set size (). (PDF) [file pcbi.1003503.s002.pdf]

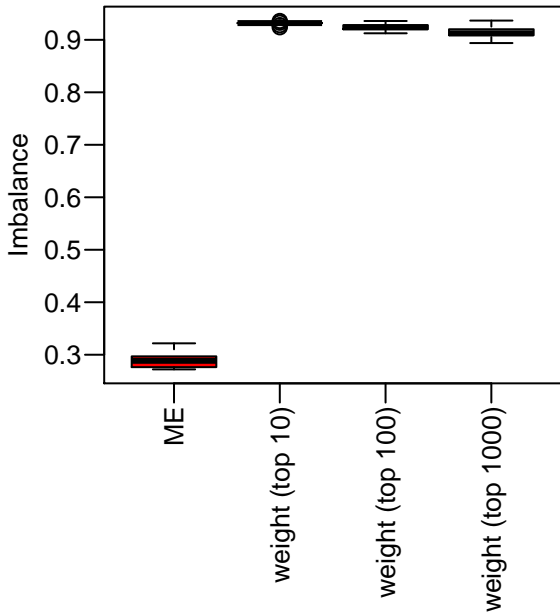

Supplement: Figure S3 — Imbalance of patterns identified with the ME approach is much lower than of patterns identified using the previously proposed weight. Box-plots summarize the imbalance distribution for 11 patterns called significant with ME p-value , high coverage () and low impurity (; red), as well as the 10, 100, and 1000 top patterns with the largest weight, called significant with permutation test (p-value ). Median imbalance of patterns prioritized using our approach is around three times lower than of patterns with top, significant weights, regardless of how many of the top ones are considered. (PDF) [file pcbi.1003503.s003.pdf]

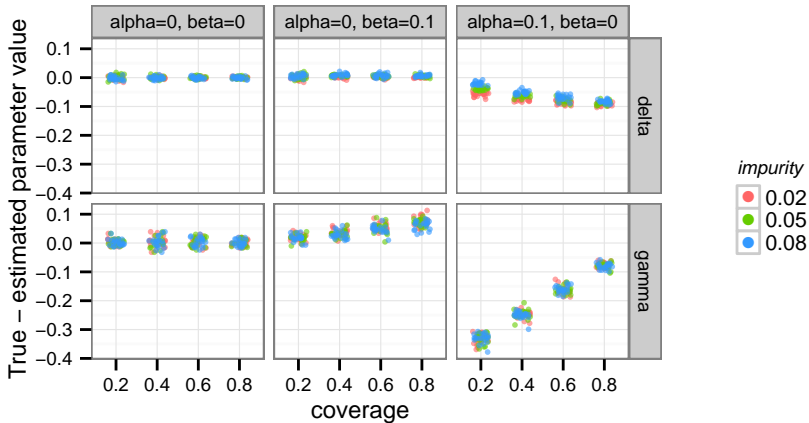

Supplement: Figure S4 — Parameter estimation in the reduced mutual exclusivity model can be severely biased by errors in the data. Left column: the difference between the true and the estimated parameter values for datasets simulated from the mutual exclusivity model without errors. In this case, both impurity (delta; top) and coverage (gamma; bottom) estimation is very accurate, regardless the impurity (marked with colors). The true coverage values are indicated on the x-axis. Middle column: addition of false negatives results in underestimation of the coverage parameter. Right column: addition of false positives results in underestimation of both the impurity and coverage parameters, and most strongly affects estimation of low coverage values. (PDF) [file pcbi.1003503.s004.pdf]

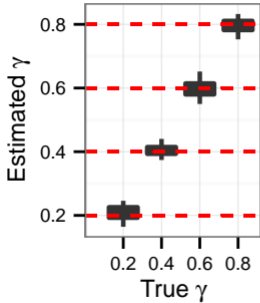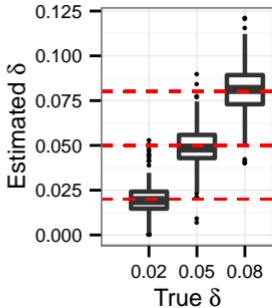

Supplement: Figure S5 — Efficient parameter estimation of the coverage parameter and the impurity parameter , using the EM algorithm, from data generated from the mutual exclusivity model with error rates that were given to the model. The tested true parameter values were fixed to , and (20 datasets with 5 genes and 1000 patients were simulated per each parameter setting). There are different box plots of estimated parameter values for different true values. The medians of the estimated values are close to the true values, marked with red dashed lines. (PDF) [file pcbi.1003503.s005.pdf]

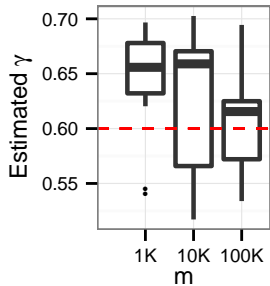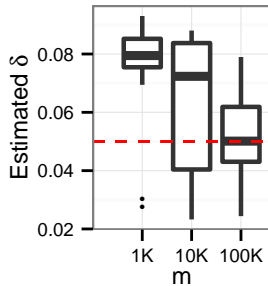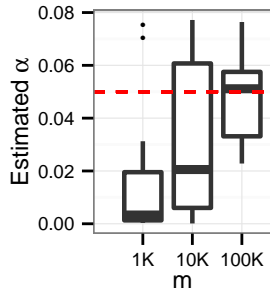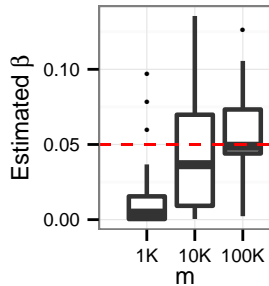

Supplement: Figure S6 — Difficulties in estimating the full set of parameters. We applied our EM algorithm to estimate the coverage parameter , the impurity parameter , as well as false positive and false negative rate , from data generated from the mutual exclusivity model with error rates that were not given to the model, using increasing sample size. The tested parameter values were fixed to realistic values , , and . 20 datasets with genes and from 1000 (1 K) to 100000 patients (100 K) were simulated. Estimation accuracy increases with sample size. (PDF) [file pcbi.1003503.s006.pdf]

A

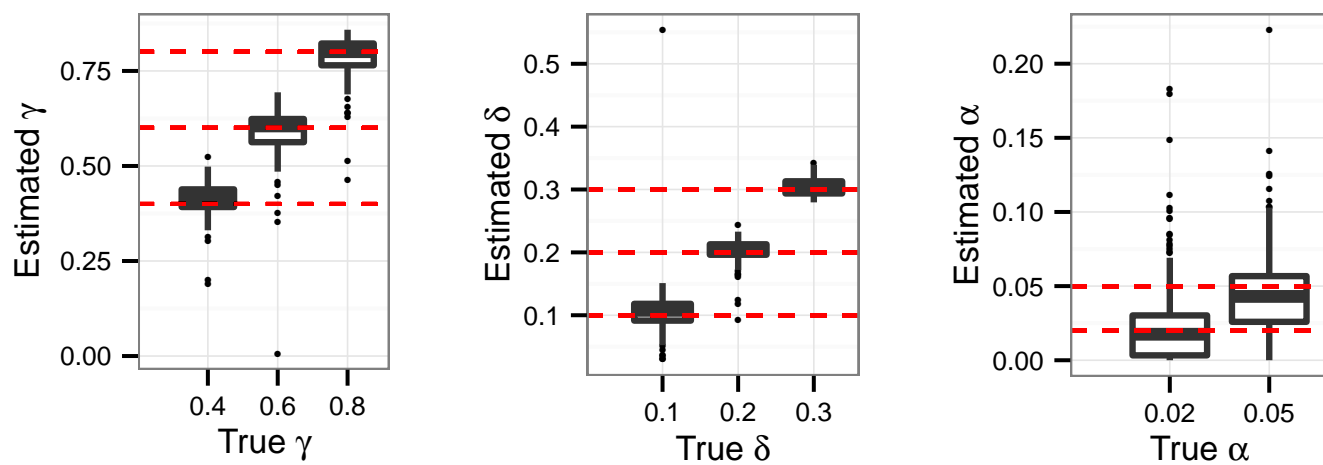

B

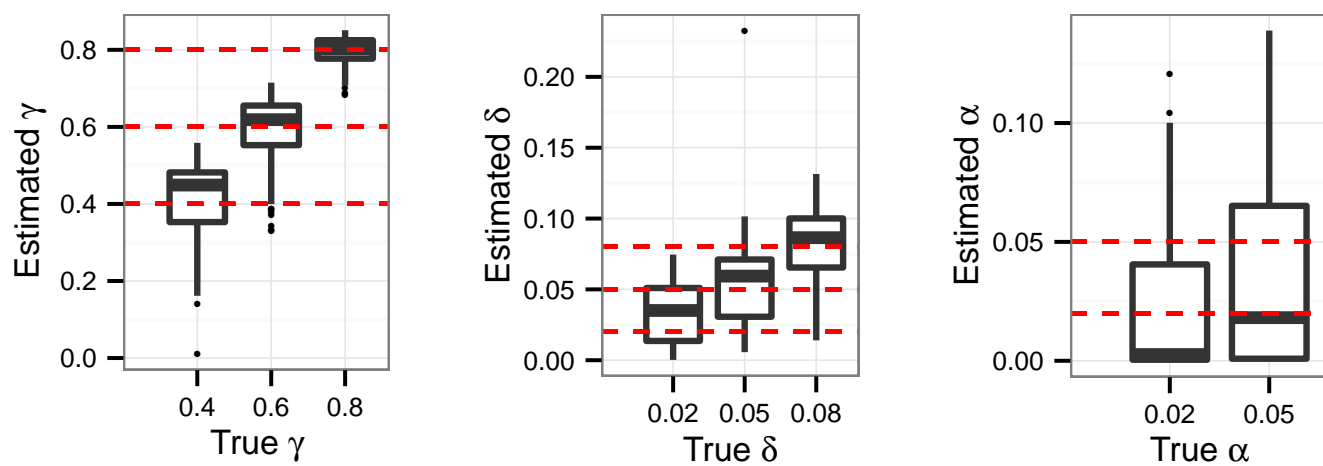

C

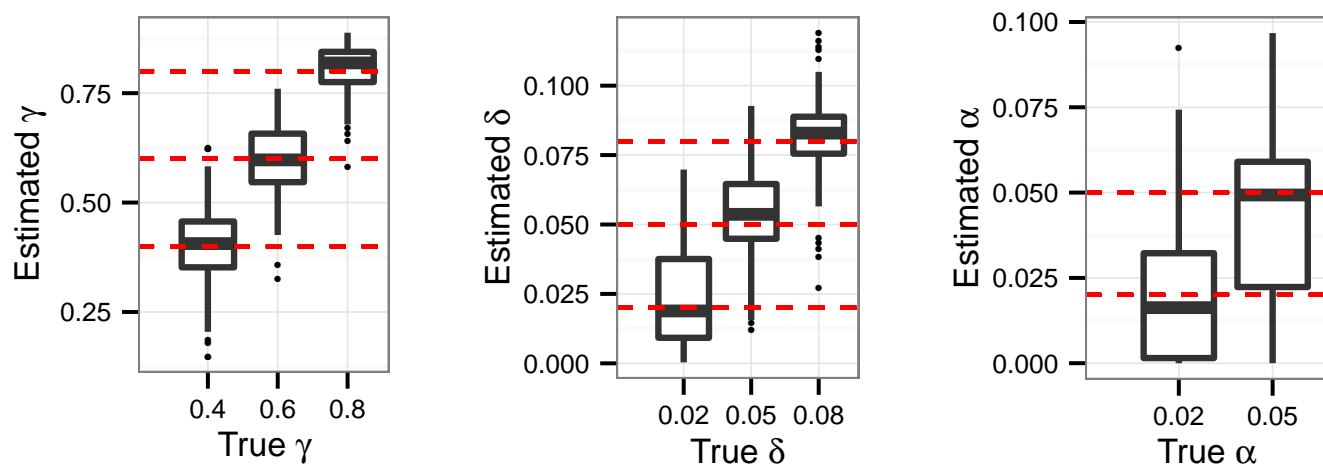

Supplement: Figure S7 — More accurate parameter estimation assuming false negative rate . A Estimation of parameters , , and from data generated from the mutual exclusivity model accounting for false positives (false positive rate was not given to the model). The tested parameter values were fixed to , , and . B The estimation is more difficult when and are similar (for ). C Similarity of and is less of a problem for larger gene sets (here, 10 genes), as well as when more samples are used (not shown). All plots: results on simulations of 20 datasets with 5 genes and 1000 patients per each parameter setting. (PDF) [file pcbi.1003503.s007.pdf]
