# Supplementary material for: Modeling Mutual Exclusivity of Cancer Mutations
Source: PLoS Comput Biol. 2014 Mar 27;10(3):e1003503. doi: 10.1371/journal.pcbi.1003503 (PMC3967923; doi:10.1371/journal.pcbi.1003503)
Supplement: Table S1 — Root mean squared error (RMSE) of parameter estimation for different model variants and sample sizes. To determine a reasonable dataset size for the different model variants, we tracked the RMSE of parameter estimates for sample sizes 200 and 1000, with typical parameter settings: , and error rates as indicated in the column “True error rates”. 20 datasets with 5 genes and the number of patients indicated in column “” were simulated from the models per each parameter setting. RMSE was chosen to represent the difficulty of the estimation task as a function of the sample size. For example, for the reduced model that assumes no errors, we have derived closed-form expressions for the maximum likelihood parameter values. Thus, in this case, RMSE of parameter estimates depends only on random variation in the data and defines the best you can get reference for the remaining models, where parameter estimation is more difficult and performed using EM. Since both the ME model likelihood and the test largely depend on how accurately the parameters are estimated, RMSE defines the applicability of the approach. (PDF) [file pcbi.1003503.s008.pdf]

Model	True error rates	m	RMSE γ	RMSE δ	RMSE α	RMSE β
Reduced, assuming $\alpha = 0$, $\beta = 0$	$\alpha = 0$, $\beta = 0$	200	0.032	0.0106	–	–
		1000	0.0145	0.0052	–	–
Known α, β	$\alpha \in (0.03, 0.06)$, $\beta \in (0.04, 0.08)$	200	0.0455	0.0227	–	–
		1000	0.01996	0.0109	–	–
Unknown α, assuming $\beta = 0$	$\alpha \in (0.02, 0.05)$, $\beta = 0$	200	0.1146	0.0515	0.0399	–
		1000	0.0815	0.0283	0.0344	–
Unknown α, β	$\alpha \in (0.03, 0.06)$, $\beta \in (0.04, 0.08)$	200	0.297	0.0692	0.0395	0.546
		1000	0.305	0.0455	0.03459	0.539
