# Supplementary material for: Modeling Mutual Exclusivity of Cancer Mutations
Source: PLoS Comput Biol. 2014 Mar 27;10(3):e1003503. doi: 10.1371/journal.pcbi.1003503 (PMC3967923; doi:10.1371/journal.pcbi.1003503)
Supplement: Table S3 — Results for mutually exclusive patterns identified in the glioblastoma dataset by previous studies. Analyzed genes are written in bold, to distinguish from genes that were filtered out in preprocessing steps. Publication: the study in which the gene set was identified as mutually exclusive. Other results are given as in Table 1. *from this gene set, only TP53 passed the pre-filtering step, and thus no results are available. (PDF) [file pcbi.1003503.s010.pdf]

Gene set	Publication	$\hat{\gamma}$	$\hat{\delta}$	ME p-value	Perm. p-value	Imbalance
CDK4, CDKN2B, RB1	[1], [2]	0.98	0.24	0.996	0	0.54
PTEN, PIK3CA, PIK3R1, IDH1, *PDPN, PRDM2*	[2]	0.98	0.11	1	0.007	0.72
TP53, *MDM2, MDM4, NLRP3, AKAP6, NPAS3* *	[2]	–	–	–	–	–
EGFR, PDGFRA, RB1	[2]	0.98	0.33	1	0.087	0.57

References

1. Vandin F, Upfal E, Raphael BJ (2012) *De Novo* discovery of mutated driver pathways in cancer. Genome Res 22: 375–385.

2. Leiserson MDM, Blokh D, Sharan R, Raphael BJ (2013) Simultaneous Identification of Multiple Driver Pathways in Cancer. PLoS Comput Biol 9: e1003054+.
