# Supplementary material for: Modeling Mutual Exclusivity of Cancer Mutations
Source: PLoS Comput Biol. 2014 Mar 27;10(3):e1003503. doi: 10.1371/journal.pcbi.1003503 (PMC3967923; doi:10.1371/journal.pcbi.1003503)
Supplement: Table S4 — Sets of genes that had identical columns in the combined pan-cancer data matrix and their short names used in the main text. Genes with identical columns in the combined and binarized pan-cancer data matrix were merged into sets and represented by a single column. The table lists those merged gene sets that are involved in top mutually exclusive patterns identified for the pan-cancer data. (PDF) [file pcbi.1003503.s011.pdf]

Merged gene set	Short name
ORAOV1, ANO1	*META 1*
POU5F1B, LOC727677, MIR1204, MIR1208, MYC, MIR1205, PVT1	*META 2*
IFNA6, KLHL9, IFNE, CDKN2B, IFNA13, DMRTA1, MIR31, IFNA2, C9orf53, IFNA8, IFNA22P, MTAP, IFNA5, IFNA1, CDKN2B-AS1	*META 3*
