# Supplementary material for: Modeling Mutual Exclusivity of Cancer Mutations
Source: PLoS Comput Biol. 2014 Mar 27;10(3):e1003503. doi: 10.1371/journal.pcbi.1003503 (PMC3967923; doi:10.1371/journal.pcbi.1003503)
Supplement: Table S5 — List of high quality, significant gene sets of size five identified in the pan-cancer dataset. (PDF) [file pcbi.1003503.s012.pdf]

Gene set	$\hat{\gamma}_0$	$\hat{\delta}_0$	ME_0 p-value	$\hat{\gamma}$	$\hat{\delta}$	$\hat{\alpha}$	ME p-value
ACTN2, EGFR, META 1, VHL, APC	0.39	0.013	10^{-4}	0.34	0	0.014	8×10^{-5}
META 2, VHL, CDKN2A, PTEN, APC	0.55	0.034	0.00518	0.45	0.001	0.037	0.00422
META 2, VHL, META 3, PTEN, APC	0.51	0.032	9×10^{-5}	0.51	0.031	0.001	7×10^{-5}
