# Supplementary material for: Modeling Mutual Exclusivity of Cancer Mutations
Source: PLoS Comput Biol. 2014 Mar 27;10(3):e1003503. doi: 10.1371/journal.pcbi.1003503 (PMC3967923; doi:10.1371/journal.pcbi.1003503)
Supplement: Table S7 — Average runtime of the EM algorithm in CPU seconds. The table presents average runtimes of parameter estimation using the EM algorithm averaged over the datasets simulated and summarized in Table S1. The runtime increases with the difficulty of the parameter estimation problem. (PDF) [file pcbi.1003503.s014.pdf]

Model	average EM runtime
Given error rates α and β	1.242621
Given $\beta = 0$, α unknown	93.23118
β and α unknown	102.0186
