# Supplementary material for: Modeling Mutual Exclusivity of Cancer Mutations
Source: PLoS Comput Biol. 2014 Mar 27;10(3):e1003503. doi: 10.1371/journal.pcbi.1003503 (PMC3967923; doi:10.1371/journal.pcbi.1003503)
Supplement: Text S1 — Supplementary Methods. Likelihood in the mutual exclusivity model, identifiability of the mutual exclusivity model, and derivation of the Expectation Maximization algorithm. (PDF) [file pcbi.1003503.s015.pdf]

Text S1
Modeling mutual exclusivity of cancer mutations

Ewa Szczurek[1], Niko Beerenwinkel[1,*]
1 Department of Biosystems Science and Engineering, ETH Zurich, Basel, Switzerland; SIB Swiss Institute of Bioinformatics
∗ E-mail: niko.beerenwinkel@bsse.ethz.ch

Likelihood in the mutual exclusivity model

The complete data likelihood of a given observation $\mathbf{y} = (y_1, ..., y_n)$ in the mutual exclusivity model, given the parameters, factorizes according to conditional independencies in the model:

$$P(\mathbf{y}, C, H, T|\theta) = \gamma^C(1-\gamma)^{1-C}\frac{1}{n}\prod_g \left((0^{1-T_g})^{CH_g}(\delta^{T_g}(1-\delta)^{1-T_g})^{C(1-H_g)}(0^{T_g})^{1-C}\epsilon(y_g, T_g)\right) \quad (1)$$

Here, for convenience, the mutually mutated gene is specified by a vector of binary random variables $H = (H_1, \ldots, H_n)$, only one of which can be assigned value 1 at a time: $P(H_g = 1) = \frac{1}{n}$, and $H_g = 1$ implies that $H_{g'} = 0$ for all $g' \neq g$.

To obtain $P(\mathbf{y}|\theta)$, the observed likelihood for observation $\mathbf{y}$ (equation 1 in the main text), we need to marginalize the hidden variables out. This likelihood depends only on the number k of values 1 in this observation, its length n, and on the parameters θ, and will be shortly denoted $f_\theta(k, n)$. Let $d = \delta(1-\beta) + (1-\delta)\alpha$.

$$\begin{aligned} f_\theta(k,n) &= \sum_c \sum_h \sum_{\mathbf{t}} P(C=c)P(H=h)P(\mathbf{y},\mathbf{t}|C=c,H=h,\theta) \quad (2) \\ &= (1-\gamma)\prod_g \sum_{t_g} P(y_g|t_g)P(t_g|C=0) + \frac{\gamma}{n}\sum_{g'}\prod_g\sum_{t_g} P(y_g|t_g)P(t_g|C=1,H_{g'}=1) \\ &= (1-\gamma)\alpha^k(1-\alpha)^{n-k} + \frac{\gamma}{n}d^{k-1}(1-d)^{n-k-1}(k(1-\beta)(1-d) + (n-k)\beta d). \end{aligned}$$

Thus, knowing k, the observed likelihood for one observation can be computed in constant time, which is possible since we assumed $P(H_g = 1) = \frac{1}{n}$. Parametrizing the distribution of H, for example by allowing parameters $p_g = P(H_g = 1)$, with $\sum_g p_g = 1$, would increase the complexity of computing this likelihood to $O(n)$. The likelihood value would no longer only depend on the number k of non-zero values, but also on which entries in the observation were non-zero. Consequently, computation of the observed likelihood of the entire dataset, now requiring initial mn pre-computing steps and $n+1$ steps of constant time complexity (equation 2 in the main text), would change its complexity to $O(mn)$. This is important for the EM algorithm, which performs the initial pre-computation once, and the likelihood is computed for all iterations in $O(n+1)$.

Identifiability of the mutual exclusivity model

We first formally prove that the four model parameters in $\theta = \{\gamma, \delta, \alpha, \beta\}$ are identifiable from the data.

Proposition 1 For $n \geq 3$, the parameters in the mutual exclusivity model are identifiable.

Proof. Consider a mapping from the parameter space Θ to the probability simplex Δ defined by the probabilities $P(\mathbf{y}|\theta)$ for all possible observations $\mathbf{y}$ (equation 2 above). We need to show that this mapping is invertible.

We construct the Jacobian matrix with columns corresponding to the four parameters in θ, and rows to all possible observations. There are $n+1$ groups of identical rows, one group per the number of values 1 in the observations in this group, denoted k. Thus, already with $n \geq 3$, the Jacobian has at least 4 unique rows. Each unique row is of the form

$$[\frac{\partial f_\theta(k,n)}{\partial \gamma}, \frac{\partial f_\theta(k,n)}{\partial \delta}, \frac{\partial f_\theta(k,n)}{\partial \alpha}, \frac{\partial f_\theta(k,n)}{\partial \beta}],$$

with the individual entries defined by:

$$\frac{\partial f_\theta(k,n)}{\partial \gamma} = -\alpha^k(1-\alpha)^{n-k} + \frac{1}{n}d^{k-1}(1-d)^{n-k-1}(k(1-\beta)(1-d)+(n-k)\beta d),$$

$$\frac{\partial f_\theta(k,n)}{\partial \delta} = \frac{\gamma}{n}(1-\alpha-\beta)d^{k-2}(1-d)^{n-k-2}\big(k(1-\beta)(1-d)(k-1-dn+d)+(n-k)\beta d(k-dn+d)\big),$$

$$\begin{aligned}\frac{\partial f_\theta(k,n)}{\partial \alpha} &= (1-\gamma)\alpha^{k-1}(1-\alpha)^{n-k-1}(k-\alpha n)+ \\ &\quad \frac{\gamma}{n}(1-\delta)d^{k-2}(1-d)^{n-k-2}\big(k(1-\beta)(1-d)(k-1-dn+d)+(n-k)\beta d(k-dn+d)\big),\end{aligned}$$

$$\frac{\partial f_\theta(k,n)}{\partial \beta} = \frac{\gamma}{n}(1-\delta)d^{k-2}(1-d)^{n-k-2}\big(d(1-d)(nd-k)-k(1-\beta)\delta(1-d)(k-1-dn+d)-(n-k)\beta\delta d(k-dn+d)\big).$$

To prove that this Jacobian is full rank, we only need to show that any of its four by four sub-matrices is of rank four. We choose the sub-matrix with simple expressions for the partial derivatives, by selecting four unique rows in the Jacobian, with values k equal to $0, 1, n-1$, and n, respectively. For those values, many of the terms in the above equations cancel out. The resulting sub-matrix

$$\begin{bmatrix} \frac{\partial f_\theta(0,n)}{\partial \gamma} & \frac{\partial f_\theta(0,n)}{\partial \delta} & \frac{\partial f_\theta(0,n)}{\partial \alpha} & \frac{\partial f_\theta(0,n)}{\partial \beta} \\ \frac{\partial f_\theta(1,n)}{\partial \gamma} & \frac{\partial f_\theta(1,n)}{\partial \delta} & \frac{\partial f_\theta(1,n)}{\partial \alpha} & \frac{\partial f_\theta(1,n)}{\partial \beta} \\ \frac{\partial f_\theta(n-1,n)}{\partial \gamma} & \frac{\partial f_\theta(n-1,n)}{\partial \delta} & \frac{\partial f_\theta(n-1,n)}{\partial \alpha} & \frac{\partial f_\theta(n-1,n)}{\partial \beta} \\ \frac{\partial f_\theta(n,n)}{\partial \gamma} & \frac{\partial f_\theta(n,n)}{\partial \delta} & \frac{\partial f_\theta(n,n)}{\partial \alpha} & \frac{\partial f_\theta(n,n)}{\partial \beta} \end{bmatrix}$$

has its reduced row echelon form of the identity matrix. With no zero-rows in the row echelon form we conclude that the sub-matrix is of rank four, and thus, for $n \geq 3$ and generic parameters, the whole Jacobian is of full rank, and the mapping is invertible.

Derivation of the Expectation Maximization algorithm

The complete log likelihood of the whole dataset $\mathbf{Y} = \{\mathbf{y}_1, \ldots \mathbf{y}_m\}$ in the mutual exclusivity model reads

$$\begin{aligned}
\log(P(\mathbf{Y}, C, H, T|\theta)) &= \sum_p \Big(C_p \log(\gamma) + (1 - C_p)\log(1-\gamma) - \log(n) + \\
&\sum_g \big(\log(0^{C_p H_{pg}(1-T_{pg})}) + \\
&C_p(1 - H_{pg})T_{pg}\log(\delta) + C_p(1 - H_{pg})(1 - T_{pg})\log(1-\delta) + \\
&\log(0^{(1-C_p)T_{pg}}) + \\
&T_{pg} y_{pg} \log(1-\beta) + T_{pg}(1 - y_{pg})\log(\beta) + \\
&(1 - T_{pg})y_{pg}\log(\alpha) + (1 - T_{pg})(1 - y_{pg})\log(1-\alpha)\big)\Big).
\end{aligned} \tag{3}$$

We show how to use the EM algorithm to estimate parameters in this model. In the E-step, we compute the expected values of relevant variables given the data and the parameters. First, we evaluate

$$\begin{aligned}
\overline{C_p} &= E[C_p|\mathbf{Y}, \theta] = \frac{P(C_p = 1, Y_p|\theta)}{P(Y_p|\theta)} \\
&= \frac{\gamma}{nP(Y_p|\theta)} d^{k_p - 1}(1-d)^{n-k_p-1}(k_p(1-\beta)(1-d) + (n-k_p)\beta d),
\end{aligned} \tag{4}$$

Note that since we assume that $P(H_g = 1) = \frac{1}{n}$, the nominator in equation (4) can be computed in constant time. This would not be the case if a set of parameters would describe the exclusive mutation frequencies instead, with one parameter per each gene: then, the exact placement of the mutually exclusive alteration in each observation would matter, and the hidden variable values would have to be summed out explicitly, in n steps. Remarkably, the value of $\overline{C_p}$ depends only on the number k_p of values 1 in observation p. Thus, instead of computing m values of $\overline{c_p}$ for each $p \in \{1, ..., m\}$, it suffices to compute $n + 1$ unique values, for each $k \in \{0, ..., n\}$:

$$\overline{c_k} = \frac{\gamma}{nf_\theta(k, n)} d^{k-1}(1-d)^{n-k-1}(k(1-\beta)(1-d) + (n-k)\beta d), \tag{5}$$

where the observed data likelihood $f_\theta(k, n) = P(Y_p|\theta)$ is computed using equation 2. Next, we compute

$$\overline{C_p T_{pg}} = E[C_p T_{pg}|\mathbf{Y}, \theta] = \frac{P(C_p = 1, T_{pg} = 1, Y_p|\theta)}{P(Y_p|\theta)} \tag{6}$$

This value depends only on the total number of values 1 in observation p, as well as on whether $y_{pg} = 0$, or $y_{pg} = 1$. For each $k \in \{0, ..., n\}$ we define auxiliary values $\overline{t_k^0}$, $\overline{t_k^1}$ respectively. Given that $k_p = k$ we have

$$\overline{C_p T}_{pg} = \begin{cases} \overline{t_k^0} & \text{if } y_{pg} = 0, \\ \overline{t_k^1} & \text{if } y_{pg} = 1, \end{cases}$$

where

$$\overline{t_k^0} = \frac{\gamma}{nf_\theta(k, n)} \beta d^{k-1}(1-d)^{n-k-2}\big(d(1-d) + k\delta(1-\beta)(1-d) + (n-k-1)\delta\beta d\big) \tag{7}$$

$$\overline{t_k^1} = \frac{\gamma}{nf_\theta(k, n)} (1-\beta) d^{k-2}(1-d)^{n-k-1}\big(d(1-d) + (k-1)\delta(1-\beta)(1-d) + (n-k)\delta\beta d\big) \tag{8}$$

Similarly, we compute

$$\overline{C_p H_{pg}} = E[C_p H_{pg}|\mathbf{Y},\theta] = \frac{P(C_p=1, H_{pg}=1, Y_p|\theta)}{P(Y_p|\theta)} \tag{9}$$

and define auxiliary values $\overline{h_k^0}$ and $\overline{h_k^1}$ such that, for $k_p = k$,

$$\overline{C_p H}_{pg} = \begin{cases} \overline{h_k^0} & \text{if } y_{pg}=0, \\ \overline{h_k^1} & \text{if } y_{pg}=1, \end{cases}$$

where

$$\overline{h_k^0} = \frac{\gamma}{n f_\theta(k,n)} \beta d^k (1-d)^{n-k-1}, \tag{10}$$

and

$$\overline{h_k^1} = \frac{\gamma}{n f_\theta(k,n)} (1-\beta) d^{k-1} (1-d)^{n-k}. \tag{11}$$

Finally, we show that

$$\overline{T_{pg}} = E[T_{pg}|\mathbf{Y},\theta] = E[C_p T_{pg}|\mathbf{Y},\theta] = \overline{C_p T_{pg}}. \tag{12}$$

Indeed,

$$E[T_{pg}|\mathbf{Y},\theta] = P(T_{pg}=1, C_p=1|\mathbf{Y},\theta) + P(T_{pg}=1, C_p=0|\mathbf{Y},\theta) = P(T_{pg}=1, C_p=1|\mathbf{Y},\theta),$$

since by definition $P(T_{pg}=1|C_p=0)=0$. Moreover, we have

$$\overline{C_p H_{pg} T_{pg}} = E[C_p H_{pg} T_{pg}|\mathbf{Y},\theta] = E[C_p H_{pg}|\mathbf{Y},\theta] = \overline{C_p H_{pg}}, \tag{13}$$

since $P(T_{pg}=1|H_pg=1)=1$. In total, the E-step comprises computations of 6(n+1) values, namely, $f_\theta(k,n)$, $\overline{c_k}$, $\overline{t_k^0}$, $\overline{t_k^1}$, $\overline{h_k^0}$, $\overline{h_k^1}$, each for $k \in \{0,...,n\}$.

In the M-step, we estimate the parameters maximizing the expected complete likelihood, given the estimated expected values of the variables. Let $k \in \{0,...,n\}$, and q_k denote the number of observations which have exactly k entries equal 1. Denote $\overline{s_k} = k\overline{t_k^1} + (n-k)\overline{t_k^0}$, the expected number of true mutations in the observation with k observed mutations. The expected complete likelihood reads

$$\begin{aligned} E[\log(P(\mathbf{Y},C,H,T|\theta))] &= \sum_p \Big(\overline{C_p}\log(\gamma) + (1-\overline{C_p})\log(1-\gamma) - \log(n) + \\ &\quad \sum_g \big((\overline{C_p T_{pg}} - \overline{C_p H_{pg}})\log(\delta) + \\ &\quad (\overline{C_p} - \overline{C_p T_{pg}})\log(1-\delta) + \\ &\quad \overline{T_{pg}} y_{pg}\log(1-\beta) + \overline{T_{pg}}(1-y_{pg})\log(\beta) + \\ &\quad (1-\overline{T_{pg}})y_{pg}\log(\alpha) + (1-\overline{T_{pg}})(1-y_{pg})\log(1-\alpha)\big)\Big) \\ &= \sum_k q_k \big(\overline{c_k}\log(\gamma) + (1-\overline{c_k})\log(1-\gamma) + \\ &\quad (\overline{s_k} - \overline{c_k})\log(\delta) + \\ &\quad (n\overline{c_k} - \overline{s_k})\log(1-\delta) + \\ &\quad k\overline{t_k^1}\log(1-\beta) + (n-k)\overline{t_k^0}\log(\beta) + \\ &\quad k(1-\overline{t_k^1})\log(\alpha) + (n-(n-k)\overline{t_k^0})\log(1-\alpha)\big), \end{aligned} \tag{14}$$

using equations (12) and (13), and since we have

$$k\overline{h_k^1} + (n-k)\overline{h_k^0} = \overline{c_k}.$$

Maximization of the expected complete likelihood with respect to γ gives

$$\tilde{\gamma} = \frac{\sum_k q_k \overline{c_k}}{m}, \tag{15}$$

maximization with respect to δ yields

$$\tilde{\delta} = \frac{\sum_k q_k(\overline{s_k} - \overline{c_k})}{(n-1)\sum_k q_k \overline{c_k}} \tag{16}$$

Finally, maximization with respect to α and β, results in, respectively:

$$\tilde{\alpha} = \frac{\sum_k q_k k(1-\overline{t_k^1})}{mn - \sum_k q_k \overline{s_k}}, \tag{17}$$

and

$$\tilde{\beta} = \frac{\sum_k q_k (n-k)\overline{t_k^0}}{\sum_k q_k \overline{s_k}}. \tag{18}$$
